# Preparation of Optical-Based Sensors for the Determination of Cardiac Myosin-Binding Protein C

**DOI:** 10.1021/acsomega.6c03802

**Published:** 2026-06-25

**Authors:** Mert Korkmaz, Adil Denizli, Duygu Çimen

**Affiliations:** 37515 Hacettepe University, Department of Chemistry, Biochemistry Division, Ankara 06800, Turkey

## Abstract

In this study, cardiac myosin-binding protein-C (cMyBP-C) imprinted and nonimprinted poly­(2-hydroxyethyl methacrylate-N-methacryloxy-(L)-tryptophan) polymeric film-based surface plasmon resonance (SPR) sensors were prepared for the detection of cMyBP-C using the microcontact imprinting method. The surface characterization of cMyBP-C imprinted (MIP) and nonimprinted (NIP) SPR sensors was investigated using contact angle measurements and atomic force microscopy. Kinetic analyses were performed in the concentration range of 0.05–300 ng/mL, and the limits of detection and quantification were calculated as 0.019 and 0.064 ng/mL, respectively. Equilibrium, adsorption, and desorption cycles were completed for 10 min in all of the kinetic analyses. The selectivity studies of MIP SPR and NIP SPR sensors were performed using the template molecule cMyBP-C with competing molecules creatine kinase-MB (CK-MB) and cardiac troponin T (cTnT). The relative selectivity coefficients of the MIP SPR sensor for cMyBP-C/cTnT and cMyBP-C/CK-MB were found to be 3.85 and 12.64 times, respectively. The applicability of the MIP SPR sensor was determined using solutions prepared in artificial plasma samples, both in the SPR system and by enzyme-linked immunosorbent assay (ELISA).

## Graphical abstract

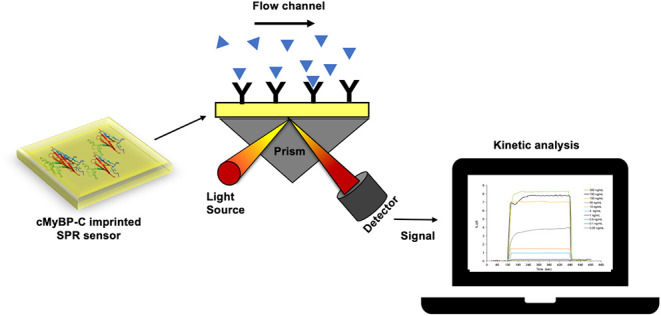

## 1 Introduction

Cardiac myosin-binding protein C (cMyBP-C) is a critical structural and regulatory protein predominantly localized within the thick filament region of cardiac muscle sarcomeres. Structurally, cMyBP-C consists of several immunoglobulin (Ig)-like and fibronectin (Fn)-like domains, which enable specific and controlled interactions with other key sarcomeric proteins, particularly myosin and titin. These interactions are crucial for the orderly function of cardiac muscle and the precise control of heart contractions. The cardiac-specific isoform of MyBP-C is uniquely characterized by distinct phosphorylation sites, which significantly influence its functional behavior throughout the contraction-relaxation cycle of the heart.^1–3^ Alterations in these phosphorylation patterns, genetic mutations, or proteolytic degradation of cMyBP-C have been closely associated with several severe cardiovascular disorders. Clinical evidence consistently links abnormalities in cMyBP-C to myocardial infarction, hypertrophic cardiomyopathy, and dilated cardiomyopathy.^4–9^ Such associations underscore the relevance and diagnostic importance of cMyBP-C as a reliable biomarker for the early identification and effective monitoring of these cardiac conditions. Clinically, accurate and timely detection of this protein could offer significant benefits in terms of understanding disease progression, providing effective patient care, and ultimately improving patient outcomes. Enzyme-linked immunosorbent assay (ELISA), immunoturbidimetry, and Western blotting are used to detect cMyBP-C.^10–12^ While these traditional tests have advantages such as high sensitivity, they also have disadvantages such as extensive sample preparation steps, time-consuming procedures, and the need for skilled personnel. To address these limitations, recent developments in sensor technology have shown promising potential.

Surface plasmon resonance (SPR) is a highly sensitive optical detection technique frequently used in the analysis of biomolecular interactions.^13^ SPR occurs when polarized light interacts with free electrons on a conductive metallic surface, commonly gold or silver, resulting in the generation of surface plasmons. SPR sensors have several critical advantages, including real-time and label-free detection, high sensitivity, rapid assay times, and suitability for kinetic analyses. SPR sensors are increasingly employed for monitoring clinical biomarkers, including nucleic acids, proteins, and small molecules, in complex biological matrices. Combining molecular imprinting technology with SPR detection methods offers a promising strategy for achieving superior selectivity and sensitivity in biomolecular sensing applications.^14–17^

Molecular imprinting technology (MIT) represents a versatile and cost-effective strategy to create synthetic molecular recognition elements that display exceptional selectivity and specificity toward chosen target molecules.^18–20^ The microcontact imprinting method is one of the surface imprinting approaches of the molecular imprinting method. In this method, the template molecule creates only selective recognition regions on the surface at the micro- or nanoscale using a “micro-stamp.” This technique has significant advantages over traditional methods, such as high activity and long-term stability of the biomolecule. Molecularly imprinted polymers (MIPs) are polymers that contain specific recognition regions specific to target molecules and are synthesized by molecular imprinting technology. In recent years, MIPs have been frequently used in various analytical and diagnostic applications, including sensor platforms for environmental monitoring, pharmaceutical analysis, and especially clinical diagnosis.^21–24^

In this study, cardiac myosin-binding protein-C (cMyBP-C) imprinted (MIP) and nonimprinted (NIP) poly­(2-hydroxyethyl methacrylate-N-methacryloxy-(L)-tryptophan) [poly­(HEMA-MATrp)] polymeric film-based surface plasmon resonance (SPR) sensors were developed using the microcontact imprinting method for cMyBP-C detection. Kinetic studies for cMyBP-C detection were performed using both aqueous solutions prepared in pH 7.4 phosphate buffer and artificial plasma. The parameters for the association kinetics analysis, equilibrium analysis (Scatchard), and different isotherm models were calculated using data obtained from kinetic analyses. The selectivity and specificity of MIP SPR sensors were investigated using creatine kinase-MB (CK-MB) and cardiac troponin T (cTnT) molecules as competing agents. cMyBP-C detection was also performed on an artificial plasma sample enriched with cMyBP-C and compared to ELISA results. Finally, the reusability of the MIP SPR sensor on the same day and at different times was tested.

## 2 Experimental Studies

### 2.1 Materials

Creatine kinase-MB (CK-MB), cardiac myosin-binding protein-C (cMyBP-C), and cardiac troponin T (cTnT) were obtained from Sigma-Aldrich (St. Louis, USA). 3-Aminopropyltriethoxysilane (APTES), 2,2’-azoisobutyronitrile (AIBN), glutaraldehyde (50%, w/v), ethylene glycol dimethacrylate (EGDMA), 2-hydroxyethyl methacrylate (HEMA), and allyl mercaptan were obtained from Fluka (Buchs, Switzerland).

### 2.2 Preparation of Protein Stamps

First, to prepare the protein stamp, glass slides measuring 24 mm × 40 mm were cleaned for 10 min with 10 mL of 1.0 M HCl, deionized water, 1.0 M NaOH, deionized water, and ethanol, respectively, and then dried with nitrogen gas. To functionalize the surfaces of the glass slides with amino groups and subsequently cross-link them with glutaraldehyde, they were incubated in a 30 mM 3-aminopropyltriethoxysilane (APTES) solution at 75 °C for 2 h. After modification, the glass slides were washed with ethanol and dried with nitrogen gas. Subsequently, they were immersed for 2 h in a 5% glutaraldehyde solution prepared in a 10 mM pH 7.4 phosphate buffer to facilitate the reaction between the amino groups on the glass slide surface.^21^ The glass slides were washed with phosphate buffer and dried with nitrogen gas, then incubated overnight at 4 °C in a 300 ng/mL cMyBP-C solution prepared in phosphate buffer (10 mM, pH 7.4) for cMyBP-C immobilization. Finally, the phosphate buffer was used to wash the glass coverslips, and they were dried using nitrogen gas. As a result, cMyBP-C immobilized protein stamps were prepared to create MIP and NIP and SPR sensors (Figure 1).

**Figure 1:**
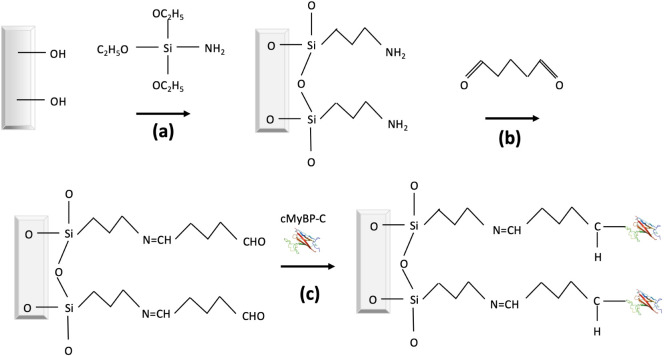
Surface modification of glass slides (**a**: glass slide modification with APTES; **b:** glass slide modification with 2.5% glutaraldehyde; **c:** immobilization of cMyBP-C).

### 2.3 Preparation of SPR Sensors

Initially, the gold-coated SPR chips underwent rigorous cleaning with piranha solution (3:1 v/v, H_2_SO_4_:H_2_O_2_) to remove organic contaminants. After being thoroughly rinsed with distilled water and dried with nitrogen, the chip surfaces were activated through the formation of a self-assembled monolayer using mercaptoethanol.

N-Methacryloyl-l-tryptophan (MATrp) monomer was synthesized and reported by Denizli and colleagues.^25^ It was selected as a monomer compatible with the template molecule cMyBP-C. The recipe for preparing cMyBP-C imprinted (MIP) and nonimprinted (NIP) poly­(HEMA-MATrp) polymeric film-based SPR sensor surfaces is as follows: Before contacting the prepared protein stamp with allyl-modified SPR chips, a polymerization mixture containing 0.6 mmol MATrp as the monomer, 0.2 mmol 2-hydroxyethyl methacrylate (HEMA) as the functional monomer, and 0.4 mmol ethylene glycol dimethacrylate (EGDMA) as the cross-linker was prepared. Four mg of 2,2’-azoisobutyronitrile (AIBN) was added to this polymerization solution as an initiator and mixed. Four μL of this mixture was taken and dropped onto the mercaptan-modified SPR chip surface to bring it into contact with the protein stamp. SPR chips bonded with a protein stamp were exposed to 100 W, 365 nm UV light for 30 min. The protein stamp was then removed from the SPR chip surface. The MIP SPR sensor was cleaned with 10 mM pH 7.4 phosphate buffer and dried with nitrogen gas. The MIP SPR sensor surface, prepared by the microcontact imprinting method, is schematically shown in Figure 2. The NIP SPR sensor was prepared identically to the imprinted ones but without the addition of cMyBP-C. The NIP SPR sensor was used to demonstrate the efficiency and specificity of the molecular imprinting process.

**Figure 2:**
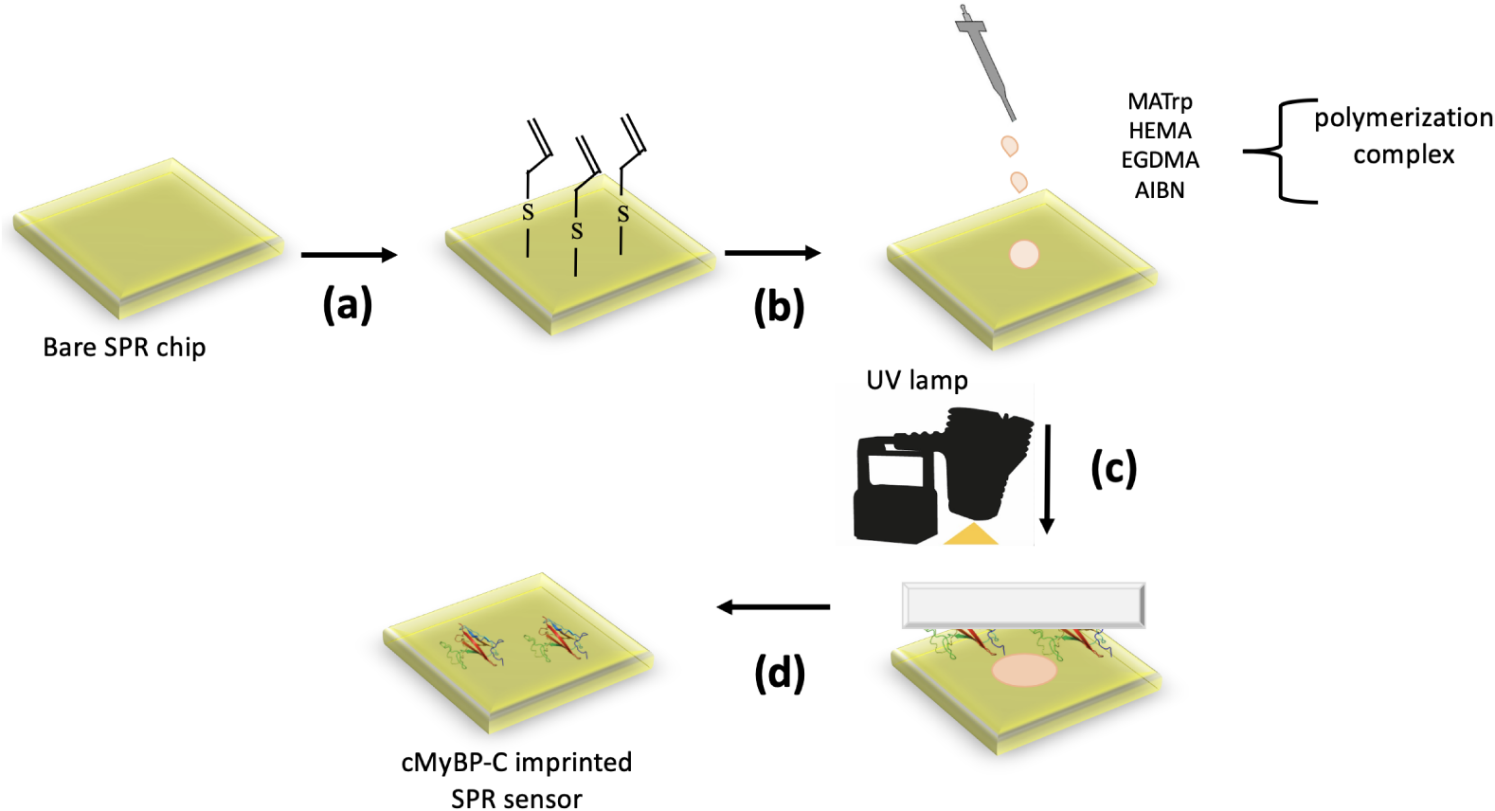
Preparation of SPR Chip Surface (**a**: modification of bare gold SPR chip surface with allyl mercaptan; **b:** dropping of polymerization solution onto the surface of the SPR chip modified with allyl mercaptan; **c:** bonding the modified glass slide to the SPR chip surface; **d:** UV polymerization).

### 2.4 Characterization Studies

The surfaces of bare gold SPR chips, MIP, and NIP SPR sensors were characterized using atomic force microscopy (AFM), contact angle measurements, and Fourier transform infrared spectroscopy by attenuated total reflectance (FTIR-ATR). The surface roughness of the bare gold SPR chip, MIP, and NIP SPR sensors was examined using AFM (NanoMagnetics Instruments). First, the SPR chips were attached to the sample holder using double-sided carbon tape, and then imaging was performed in air at a resonance frequency of 341.30 kHz in half mode. Imaging of the SPR chip surfaces was performed at a resolution of 256 × 256 pixels, over an area of 2 × 2 μm^2^, and at a scanning speed of 2 μm/s.

As a second characterization method, the hydrophilicity of SPR sensor surfaces was investigated by measuring contact angles using the fixed drop method with a KRUSS DSA100 (Hamburg, Germany) device. For this purpose, pure water was dropped onto different areas of the SPR chip surfaces, and photographs taken in various areas were used to measure the contact angles at each location. The average of five separate measurements was calculated using DSA2 software to determine the contact angles of the bare gold SPR chip, MIP, and NIP SPR sensor surfaces. FTIR-ATR spectra of MIP and NIP poly­(HEMA-MATrp) polymeric film-based SPR sensor surfaces were examined in the range 600–4000 cm^–1^ using a Fourier Transform Infrared Spectrometer (Thermo Fisher Scientific, Nicolet iS10, Waltham, USA)

### 2.5 Kinetic Analysis

All kinetic analyses were carried out using an SPRimager II system (GWC Technologies, Madison, USA). In the kinetic analyses, first, 10 mM pH 7.4 phosphate buffer was passed through the SPR system as an equilibration buffer at a flow rate of 150 μL/min for 2 min. For cMyBP-C determination, aqueous cMyBP-C solutions prepared in 10 mM pH 7.4 phosphate buffer at concentrations ranging from 0.05 ng/mL to 300 ng/mL were introduced through the SPR system for 6 min each and recorded in real-time. Then, a 10% ethylene glycol solution was introduced into the SPR system as a desorption solution for 2 min to remove cMyBP-C molecules bound to the SPR sensor surface. The analysis time was optimized to 10 min in all kinetic analyses. After each desorption step, the SPR system was washed with 10 mM pH 7.4 phosphate buffer for 2 min to prepare it for the next kinetic analysis. SPR sensorgrams obtained from kinetic analyses are plotted as the change in the refractive index of light over time (%ΔR). Furthermore, parameters for equilibrium and kinetic isotherm models were calculated using data obtained from kinetic analyses performed with the MIP SPR sensor.

The specificity and selectivity of the MIP SPR sensor against the cMyBP-C molecule were determined by selecting cardiac troponin T (cTnT, MW: 37 kDa) and creatine kinase-MB (CK-MB, MW: 84 kDa) as competing proteins, as they resemble cMyBP-C (MW: 140 kDa) molecules in terms of molecular weight, shape, and size. First, cTnT and CK-MB proteins, prepared at a concentration of 100 ng/mL in pH 7.4 phosphate buffer, were added separately to the SPR system. Subsequently, a cTnT+CK-MB binary mixture and a cMyBP-C+cTnT+CK-MB ternary mixture, each at a concentration of 100 ng/mL, were added to the SPR system and recorded in real time. Kinetic analyses were also performed with the NIP SPR sensor to observe the effect of molecular imprinting. Using the values obtained from kinetic analyses for selectivity, the selectivity coefficient (k: ΔR_template_/ΔR_competitor_) and relative selectivity coefficients (k′: k_MIP_/k_NIP_) were calculated using the following equations.

One of the most important advantages of the MIP SPR sensor prepared by MIT is its reusability. For this purpose, kinetic analyses involving the adsorption–desorption-regeneration cycle with a 100 ng/mL concentration of cMyBP-C solution were performed four times consecutively using the same chip. The kinetic analysis of the cMyBP-C solution using the same chip was conducted to investigate the shelf life and performance of the MIP SPR sensor at different times, such as the first, second, fourth, and sixth months.

### 2.6 Detection of cMyBP-C in Artificial Plasma Samples

Artificial plasma samples were used for kinetic analyses involving solutions with different concentrations of cMyBP-C. For this purpose, solutions containing 50 ng/mL and 100 ng/mL cMyBP-C were prepared in artificial plasma solutions. First, the SPR system was equilibrated by passing a pH 7.4 phosphate buffer through it for 2 min. Then, the 50 ng/mL and 100 ng/mL cMyBP-C solutions prepared in artificial plasma were separately introduced into the SPR system for 6 min. Finally, SPR sensorgrams obtained by passing the 10% ethylene glycol solution, which is the desorption solution, through the SPR system for 2 min were recorded in real time. The kinetic analysis results from the SPR sensor were compared to those of ELISA.

## 3 Results and Discussion

### 3.1 Surface Characterization

A KRUSS DSA100 instrument was utilized to measure the contact angles of bare gold SPR chip surfaces, as well as MIP SPR and NIP SPR sensor surfaces, through the sessile drop method. A drop of water was applied to all chip surfaces, and the average of 5 different contact angle measurements was calculated. The contact angles of the bare gold SPR chip surface, MIP SPR, and NIP SPR sensor surfaces were measured as 79.1°, 83.3°, and 82.4°, respectively (Figure 3). The template molecule cMyBP-C and MATrp monomer in the polymeric film structure on MIP and NIP SPR sensor surfaces possess hydrophobic properties. For this reason, the contact angles of MIP and NIP SPR sensor surfaces increased compared to the bare gold SPR chip surface. These results confirm the effectiveness of surface modifications and the successful imprinting of cMyBP-C onto the surface. The higher contact angle of the MIP SPR sensor surface has increased the surface’s ability to repel water, which indicates a more pronounced hydrophobic character.^12,26^

**Figure 3:**
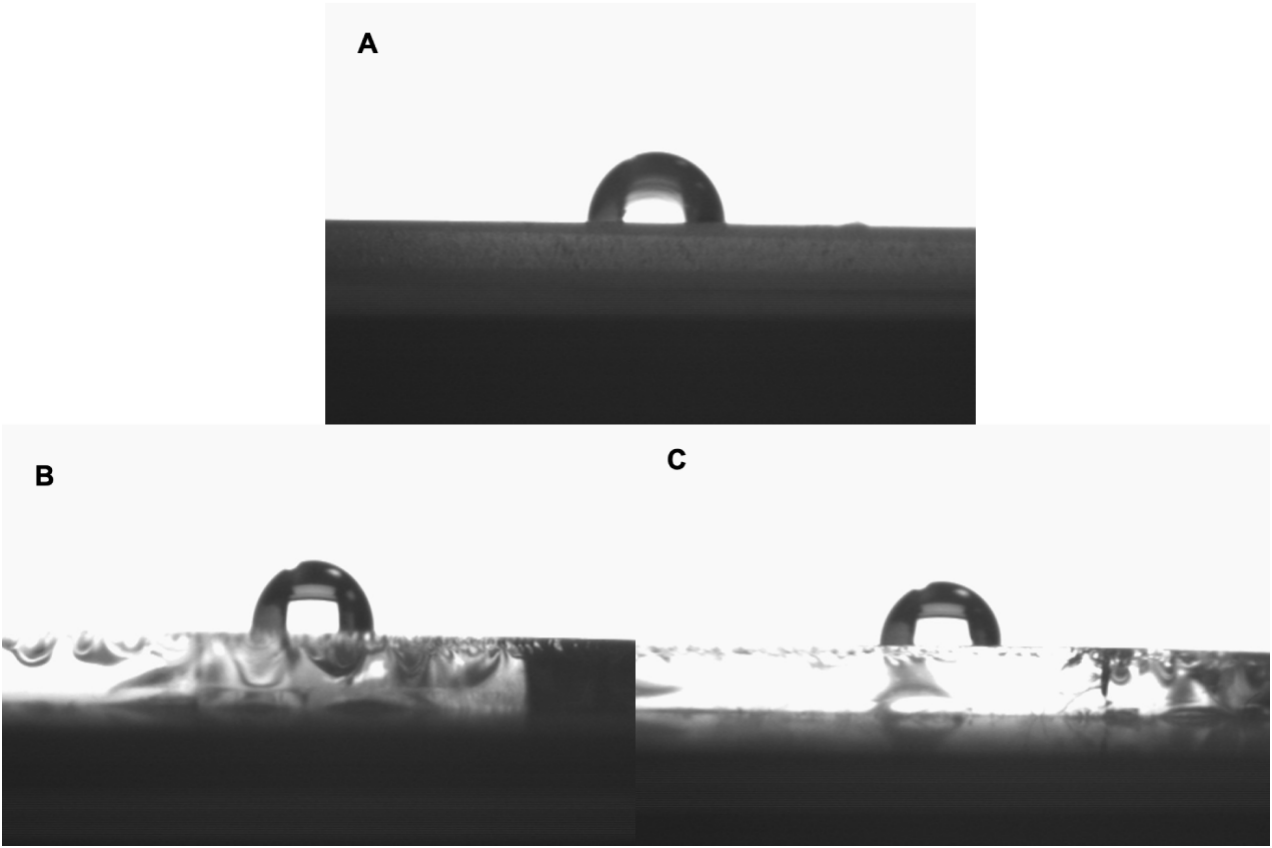
Contact angle images of (A) bare gold surface, (B) MIP SPR, and (C) NIP SPR sensor surfaces.

The surface roughness of the bare gold SPR chip surface, MIP SPR, and NIP SPR sensor surfaces was characterized using an AFM device. The surface roughness of the bare gold SPR chip surface, MIP SPR, and NIP SPR sensor surfaces was found to be 8.23, 113.94, and 90.34 nm, respectively. Examination of the AFM results showed that cMyBP-C imprinting on the modified SPR chip surfaces was successfully achieved (Figure 4).

**Figure 4:**
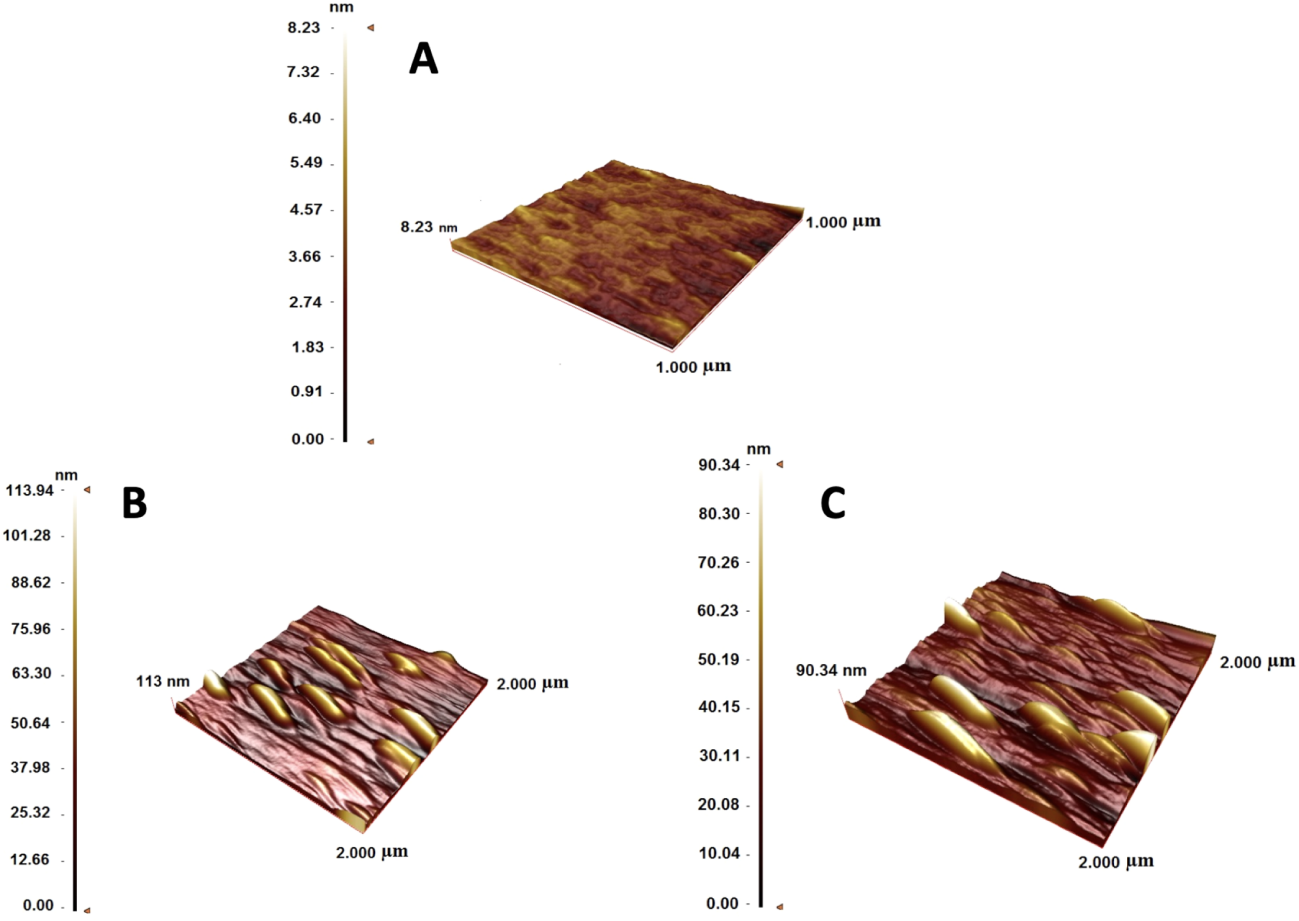
AFM images (bare gold SPR chip surface (A), MIP SPR (B), and NIP SPR (C) sensor surfaces).

When the FTIR-ATR spectra are examined, the bands observed in both spectra in the region of approximately 2918–2941 cm^–1^ correspond to aliphatic C–H stretching vibrations. The presence of these bands in both structures indicates that the structure of the poly­(HEMA-MATrp) polymeric film is preserved. The strong peaks observed around 1721–1726 cm^–1^ confirm the characteristic vibrations of ester carbonyl (C=O) groups and the ester functionality in the HEMA structure. The increased prominence of this band in the polymeric film on the MIP SPR sensor surface indicates the development of hydrogen bonding and/or specific molecular interactions between cardiac myosin-C and the polymer film. The band clearly observed around 1657 cm^–1^ on the polymeric film of the MIP SPR sensor corresponds to the amide I band of proteins and originates from C = O stretching vibrations. Similarly, the band around 1532 cm^–1^ can be associated with amide II vibrations (N–H bending and C–N stretching). The presence of amide I and amide II bands is particularly important for confirming protein imprinting. The bands observed in the range of 1449–1367 cm^–1^ are associated with CH_2_ bending and C–N vibrations. In the spectrum of the polymeric film on the NIP SPR sensor surface, C–O vibrations are particularly observed around 1148 and 1033 cm^–1^. However, the absence of amide bands confirms that protein imprinting did not occur in the polymeric film on the NIP SPR sensor surface. In conclusion, the FTIR-ATR spectra reveal that cardiac myosin-C protein was successfully imprinted into the poly­(HEMA-MATrp) polymeric film and that specific molecular interactions occurred in the polymer structure as a result of the imprinting process (Figure S1).

### 3.2 Kinetic Analysis of cMyBP-C Binding

Kinetic analyses for cMyBP-C detection were performed using MIP SPR and NIP SPR sensors prepared for this purpose, with aqueous cMyBP-C solutions at concentrations of 0.05–300 ng/mL prepared in pH 7.4 phosphate buffer, using the SPRimager II system. First, the MIP SPR sensor was equilibrated with a pH 7.4 phosphate buffer solution for 2 min, and then cMyBP-C solutions at concentrations ranging from 0.05 to 300 ng/mL were introduced into the SPRimager II system for 6 min. Finally, a 10% ethylene glycol solution was used for 2 min to remove cMyBP-C molecules from the MIP SPR sensor surface. When the data obtained for the MIP SPR sensor in the 0.05–50 ng/mL cMyBP-C concentration range were examined, the equation of the resulting line was y = 0.0776x + 0.3017, and the coefficient of linearity (R^2^) was 0.9088, while for the data obtained in the 100–300 ng/mL cMyBP-C concentration range, the equation of the resulting line was y = 0.0054x + 6.73, and the coefficient of linearity (R^2^) was 0.871 (Figure 5B).

**Figure 5:**
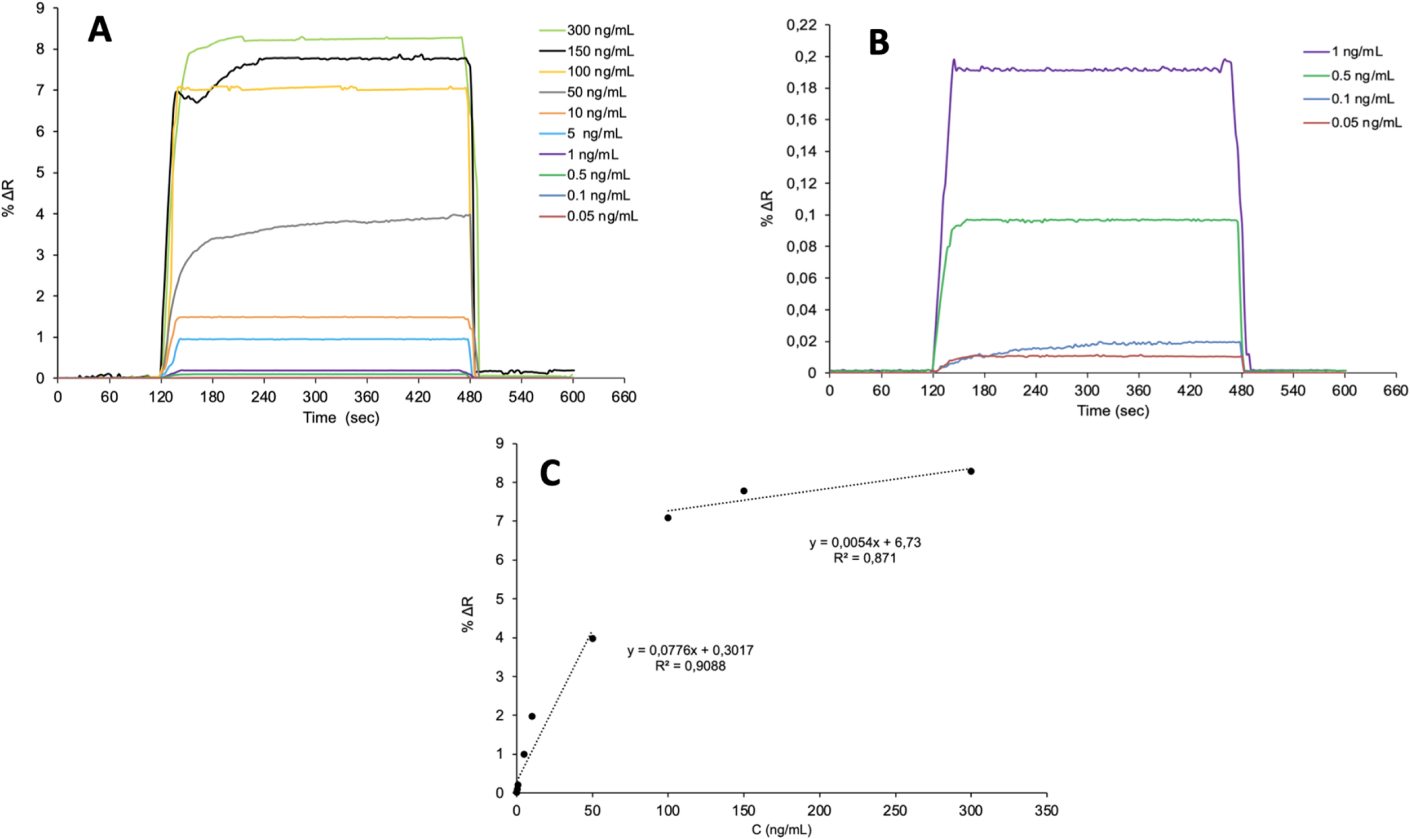
Kinetic analysis (real-time detection at different cMyBP-C concentrations (A: 0.05–300 ng/mL and B: 0.05–1 ng/mL) and concentration dependency (C) of the MIP SPR sensor, n: 3).

The limit of detection (LOD) and limit of quantification (LOQ) values were calculated from the kinetic analysis data. The LOD was calculated using the 3S/m equation, while the LOQ was determined using the 10S/m equation. S is the standard deviation of the intercept, and m is the slope of the regression line. The equilibrium-buffer response (ΔR) value was calculated using the equilibrium buffer and by averaging five measurements, with a standard deviation of 0.0005. Also, these calculations were performed using an MIP SPR sensor chip. The LOD and LOQ were calculated as 0.019 ng/mL and 0.064 ng/mL, respectively, using the equation y = 0.0776x + 0.3017 for the calibration graph.

There are very few sensor-based studies on cMyBP-C detection in the literature, and these studies are summarized in Table 1.

**Table 1:** A Summary of Sensor-Based Studies on cMyBP-C Detection in the Literature

Sensor Type	Linear Range	LOD	Real Sample	Referance
ELISA	-	122.44 ng/mL	plasma	^2^
ELISA	0–4 ng/mL	0.126 ng/mL	-	^10^
Immunoassay	0.2000 ng/L	0.4 ng/L	human plasma	^12^
Colorimetric/SERS	0–640 pg/mL	Colorimetric; pg/mL	serum	^27^
		SERS; 0.77 pg/mL		
SPR sensor	0.05–300 ng/mL	0.019 ng/mL	aqueous solution	This study

MIP SPR sensor kinetic analyses were used to investigate binding kinetic analyses and different isotherm models at various cMyBP-C concentrations (Figures S2 and S3). It was determined that the Langmuir adsorption isotherm model is the best model to determine cMyBP-C. According to this model, the homogeneous bond between the cMyBP-C molecule and the MIP SPR sensor is present, and there is minimal lateral interaction in the monolayer. According to the obtained constants, the theoretical ΔR_max_ value calculated in the Langmuir adsorption isotherm model (8.203) is very close to the experimental ΔRmax value (8.29). Furthermore, according to the kinetic analysis results, the binding kinetic analysis showed that the cMyBP-C molecule binds to the MIP SPR sensor surface with 99% accuracy. In addition, the ΔR_max_, R^2^, K_A_, and K_D_ values are given in Table S1 and Table 2.

**Table 2:** Selectivity and Relative Selectivity Coefficients

	MIP SPR Sensor		NIP SPR Sensor		
Molecules	ΔR	k	ΔR	k	k’
cMyBP-C	7.08	-	0.67	-	10.56
cTnT	1.84	3.85	0.73	0.92	4.18
CK-MB	0.56	12.64	0.57	1.18	10.71
cTnT+CK-MB	1.27	5.57	0.58	1.16	4.80
cMyBP-C+cTnT+CK-MB	5.97	1.19	0.60	1.12	1.06

### 3.3 Selectivity Studies

The selectivity of the MIP SPR sensor was investigated using binary (cTnT+CK-MB) and ternary (cMyBP-C+cTnT+CK-MB) solutions with cardiac troponin T (cTnT) and creatine kinase-MB (CK-MB) molecules separately, against the cMyBP-C molecule. The NIP SPR sensor was prepared to compare the selectivity of the MIP SPR sensor, and selectivity kinetic analyses were performed using the NIP SPR sensors. As seen in Figure 6A, the response of the MIP SPR sensor to cMyBP-C molecules was observed to be higher than that to cTnT and CK-MB molecules. This is due to the selective vacancies for cMyBP-C molecules formed on the surface of the cMyBP-C imprinted polymeric film. As shown in Table 2, k’ results demonstrated that the MIP SPR sensor has higher selectivity for cMyBP-C compared to cTnT and CK-MB. k’ of MIP SPR sensor for cMyBP-C/cTnT and cMyBP-C/CK-MB were found to be 3.85- and 12.64-fold, respectively. A relative selectivity value of k’ ≥ 1 indicates that the MIPs exhibit a higher affinity for the target molecule compared to the NIPs.^28^ This also confirms that specific recognition gaps were successfully formed during the imprinting process. Examining the selectivity results, it was proven that the MIP SPR sensor has higher selectivity for cMyBP-C determination than the NIP SPR sensor. The reason why the SPR signal in the MIP SPR sensor decreased from 7.08 for cMyBP-C to 5.97 for cMyBP-C+cTnT+CK-MB is due to the competition between the three molecules on the MIP SPR sensor surface. This is because only the vacancies for cMyBP-C molecules exist on the MIP SPR sensor surface. Therefore, when molecules in the ternary mixture attempt to enter the voids belonging to the cMyBP-C molecule, nonspecific interactions and differences in the molecular recognition mechanism cause the signal level to decrease. This reduction is observed to be very small when compared to the signals obtained in the single analyses of cTnT and CK-MB molecules and in the double analyses of cTnT+CK-MB. Based on these results, it can be concluded that the MIP SPR sensor recognizes the cMyBP-C molecule with good selectivity, thanks to the imprinting process that creates molecular shape and chemical recognition memory.

**Figure 6:**
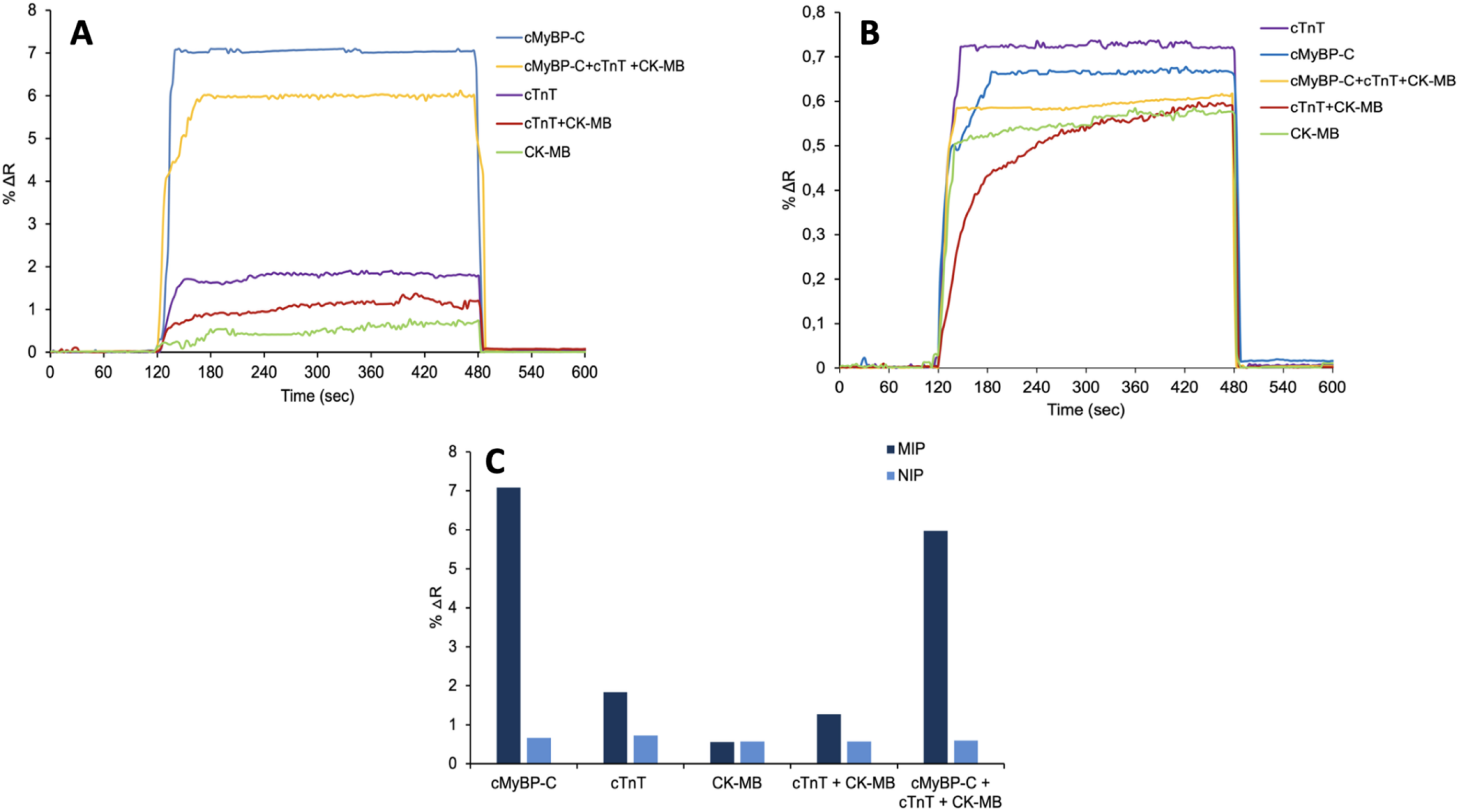
Selectivity sensorgrams of MIP (A) and NIP (B) SPR sensors, and comparison of the selectivity of MIP and NIP SPR sensors in the same graph (C) (n: 3).

Furthermore, the selectivity of the MIP SPR sensor was calculated as 10.56, with an imprinting factor (IF) determined as ΔR_MIP_/ΔR_NIP_. In summary, it was demonstrated that cMyBP-C imprinted polymeric films enhance adsorption selectivity and that certain recognition sites are not suitable for competing molecules such as cTnT and CK-MB.

### 3.4 Analysis in Artificial Plasma

The applicability of MIP SPR sensors has also been investigated in an artificial plasma environment that mimics the complex biological environment encountered in clinical diagnostic tests. MIP SPR sensors were used in kinetic analyses for the determination of cMyBP-C in artificial plasma samples. The artificial plasma solutions containing 50 ng/mL and 100 ng/mL of cMyBP-C were prepared for kinetic analysis. First, pH 7.4 phosphate buffer was passed through the SPR system for 2 min, followed by separate artificial plasma solutions with different concentrations of cMyBP-C being added for 6 min. Finally, a 10% ethylene glycol desorption solution was added to the system for 2 min, and kinetic analyses were performed in real time (Figure 7). The recovery rates obtained from 50 ng/mL and 100 ng/mL cMyBP-C solutions prepared in artificial plasma with an MIP SPR sensor were calculated as 99.42% and 97.93%, respectively (Table 3). These results highlight the robustness of the sensors and demonstrate their potential applicability in clinical settings, where complex sample matrices are common.

**Figure 7:**
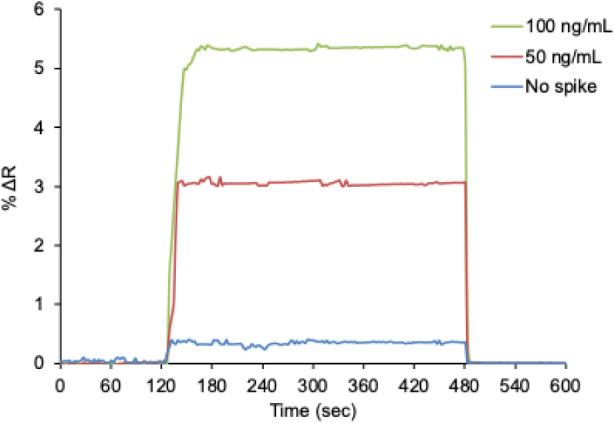
Real-time sensorgrams for cMyBP-C detection from artificial plasma samples with the MIP SPR sensor (n: 3).

**Table 3:** Analysis Results of cMyBP-C Detected in Artificial Plasma Samples

	Found cMyBP-C (ng/mL)		Recovery (%)	
Spiked cMyBP-C amount	SPR	ELISA	SPR	ELISA
50 ng/mL	49.71 ± 0.271	49.15 ± 0.092	99.42 ± 0.093	98.29 ± 0.185
100 ng/mL	97.94 ± 0.0096	97.93 ± 0.0095	97.93 ± 0.010	97.94 ± 0.010

### 3.5 Reusability Studies

The reusability of SPR sensors prepared using molecular imprinting is crucial for clinical applications. The reusability of the developed MIP SPR sensor was evaluated by conducting kinetic analyses on the same day and using the same SPR chip. The MIP SPR sensor was able to be used four times by testing it under four different concentration solutions of cMyBP-C (100 ng/mL) through equilibration, adsorption, and desorption cycles, as demonstrated in Figure 8A. After 6 months, the efficacy and efficiency of the MIP SPR sensor were also tested using the same chip and the same concentration of cMyBP-C solution (Figure 8B). After multiple adsorption cycles, the binding capacity of the MIP SPR sensor was observed to be approximately 96.46% on the same day, while at different times, the binding capacity was determined to be approximately 91%. According to the reusability results, the lack of significant change in the ΔR values of the MIP SPR sensor means that there is no significant loss in performance. These results suggest that the MIP SPR sensor can be used for a long time in clinical settings.

**Figure 8:**
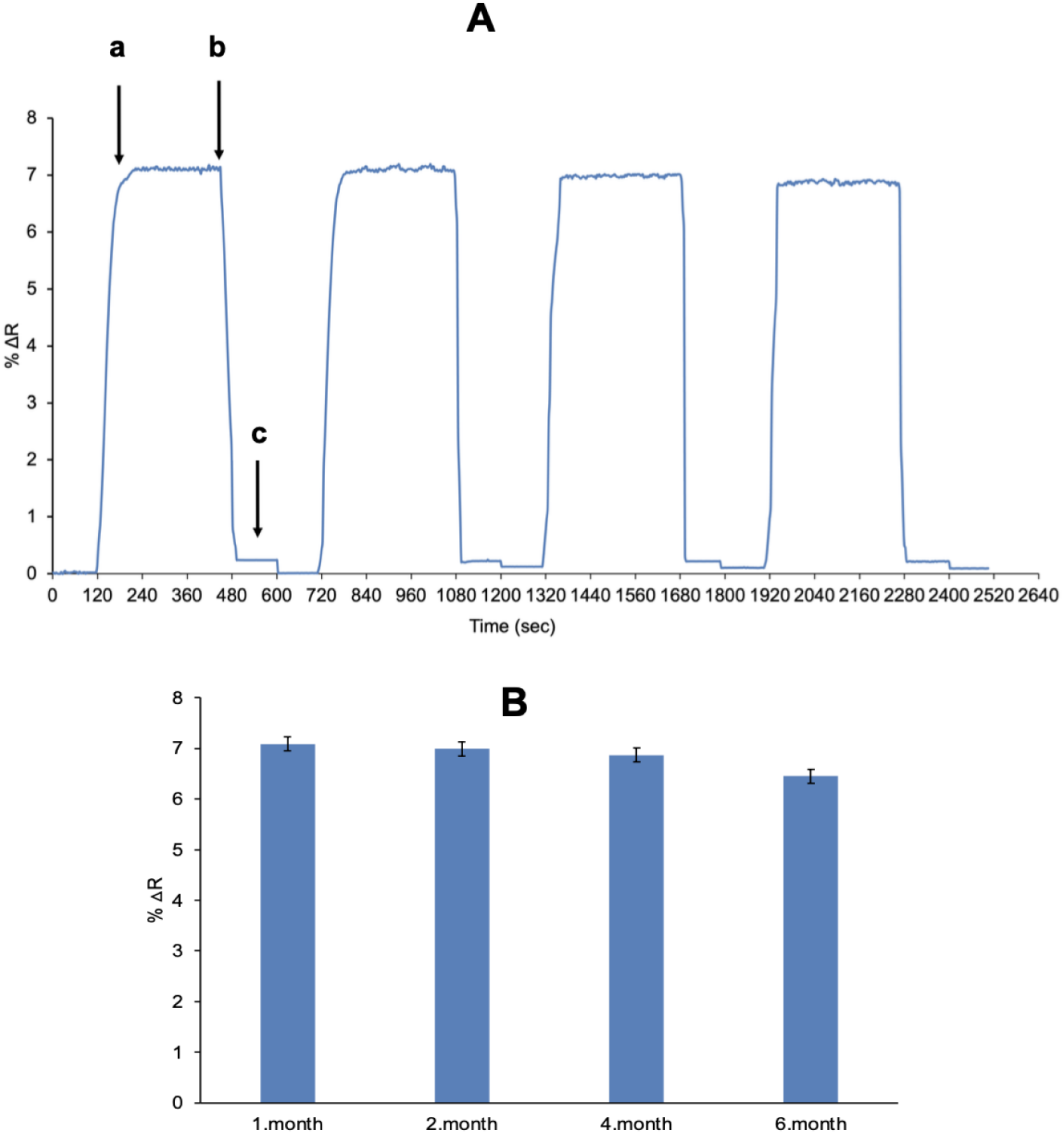
Reusability (A) and storage stability (B) of the MIP SPR sensor (a: equilibration, b: adsorption, c: desorption cycles).

## 4 Conclusions

cMyBP-C is a critical biomarker involved in the regulation of cardiac muscle contraction. Various biochemical methods are being developed for cMyBP-C detection, particularly in acute cMyBP-C cases, which provide low-cost, rapid, and accurate diagnostic results. In this study, MIP SPR and NIP SPR sensors were prepared for cMyBP-C detection in both aqueous solutions and artificial plasma samples using a microcontact imprinting method on the gold surface of the SPR sensor. The LOD and LOQ values of the MIP SPR sensor were determined as 0.019 ng/mL and 0.064 ng/mL, respectively. In selectivity studies of MIP SPR and NIP SPR sensors, cTnT and CK-MB were used as competing agents, and the imprinting factor was calculated as 10.56. Based on the Langmuir adsorption isotherm model, which is the perfect model for cMyBP-C detection, it can be concluded that the interaction between the cMyBP-C molecule and the MIP SPR sensor is homogeneous and there is minimal lateral interaction in the monolayer. The MIP SPR sensor’ reusability was not affected by a significant decrease in cMyBP-C detection performance over 4 cycles, but the initial cMyBP-C detection performance decreased to 91% after 6 months. Recovery rates of approximately 97–99% were achieved in analyses using SPR systems and ELISA methods with different concentrations of cMyBP-C solutions prepared in artificial plasma solutions. In summary, the data obtained show that the prepared MIP SPR sensor can perform selective, fast, and low detection limit analysis for detecting cMyBP-C in clinical studies.

## Supplementary Material



## Notes

Results of FTIR-ATR analysis, the binding kinetic analyses , and different isotherm models of cardiac myosin-binding protein-C imprinted poly­(2-hydroxyethyl methacrylate-N-methacryloxy-(L)-tryptophan) polymeric film-based surface plasmon resonance sensors (PDF)
