# Supplementary material for: Preparation of Optical-Based Sensors for the Determination of Cardiac Myosin-Binding Protein C
Source: ACS Omega. 2026 Jun 25;11(26):39284–92. doi: 10.1021/acsomega.6c03802 (PMC13347656; doi:10.1021/acsomega.6c03802)
Supplement: Supplementary file 1 [file ao6c03802_si_001.pdf]

PREPARATION OF OPTICAL-BASED SENSORS FOR THE DETERMINATION OF CARDIAC MYOSIN BINDING PROTEIN-C

Mert Korkmaz[1], Adil Denizli[1], Duygu Çimen[1*]

[1] Hacettepe University, Department of Chemistry, Biochemistry Division, Ankara, Turkey

FTIR-ATR spectra of cardiac myosin-binding protein-C (cMyBP-C) suppressed (MIP) and unsuppressed (NIP) poly(2-hydroxyethyl methacrylate-N-methacryloxy-(L)-tryptophan) polymeric film-based surface plasmon resonance sensor surfaces are shown in Figure S1.

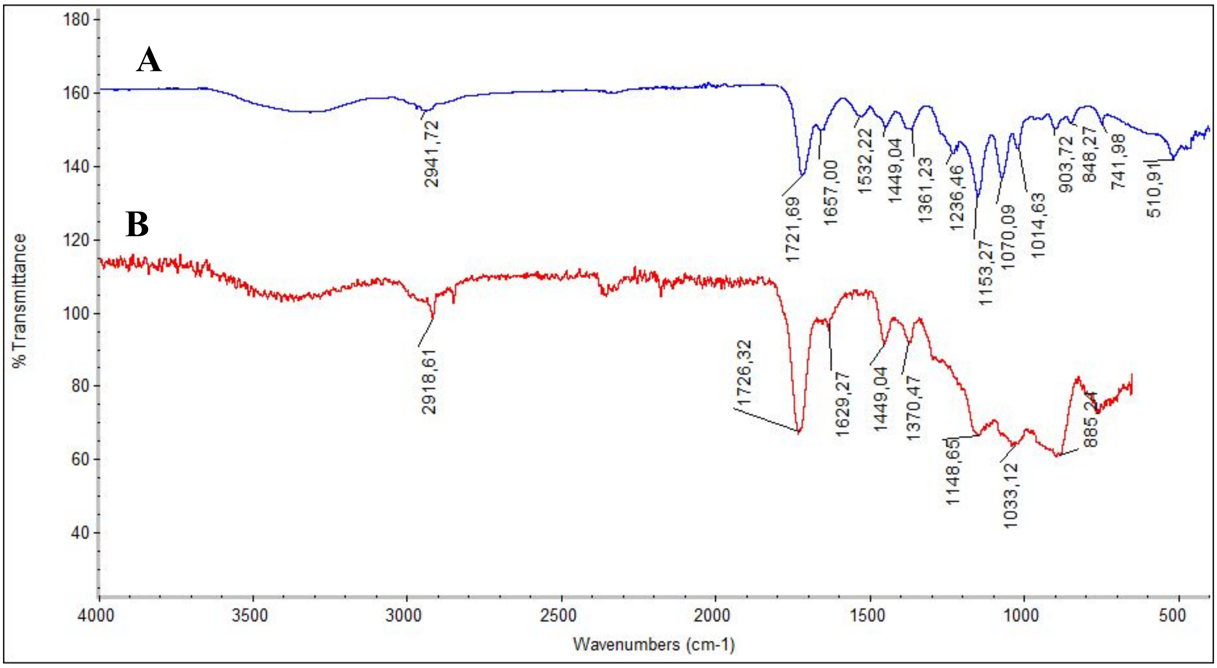

Figure S1. FTIR-ATR spectra of MIP (A) and NIP (B) SPR sensors.

The measured response (ΔR) of the SPR signal generated by the binding of cMyBP-C protein to the SPR sensor surface, cMyBP-C concentration, C (ng/mL), and equilibrium constants for binding and dissociation are expressed as K_A and K_D. Additionally, the Freundlich constant (1/n) and kinetic rate constants for binding and dissociation are also shown as k_a and k_d

Association kinetic analysis	$d\Delta R/dt = k_aC(\Delta R_{max} - \Delta R) - k_d\Delta R$	(1)
Equilibrium analysis	$\Delta R_{ex}/C = K_A(\Delta R_{max} - \Delta R_{eq})$	(2)
Langmuir	$\Delta R = (\Delta R_{max}\ C/K_D + C)$	(3)
Freundlich	$\Delta R = \Delta R_{max}\ C^{1/n}$	(4)
Langmuir-Freundlich	$\Delta R = ((\Delta R_{max}\ C^{1/n}/K_D) + C^{1/n})$	(5)

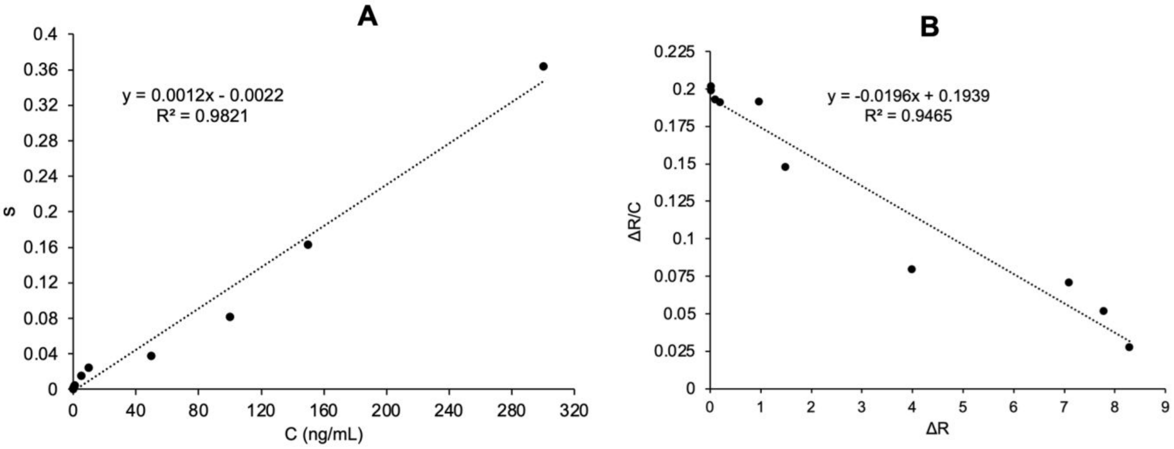

Figure S2. Determination of the kinetic binding constants between cMyBP-C molecule and MIP SPR sensor (association kinetics analysis (A) and equilibrium analysis (Scatchard) (B)).

TABLE S1. Kinetic Parameters of MIP SPR Sensor.

Equilibrium analysis (Scathard)	Association kinetics analysis
ΔR_{max}: 9.89	k_a, $(ng/mL)^{-1}.s^{-1}$: 0.0012
K_A, $(ng/mL)^{-1}$:0.0196	k_d, s^{-1}: 0.0022
K_D, ng/mL: 51.02	K_A, $(ng/mL)^{-1}$: 0.545
R^2: 0.9465	K_D, ng/mL: 1.833
	R^2: 0.9821

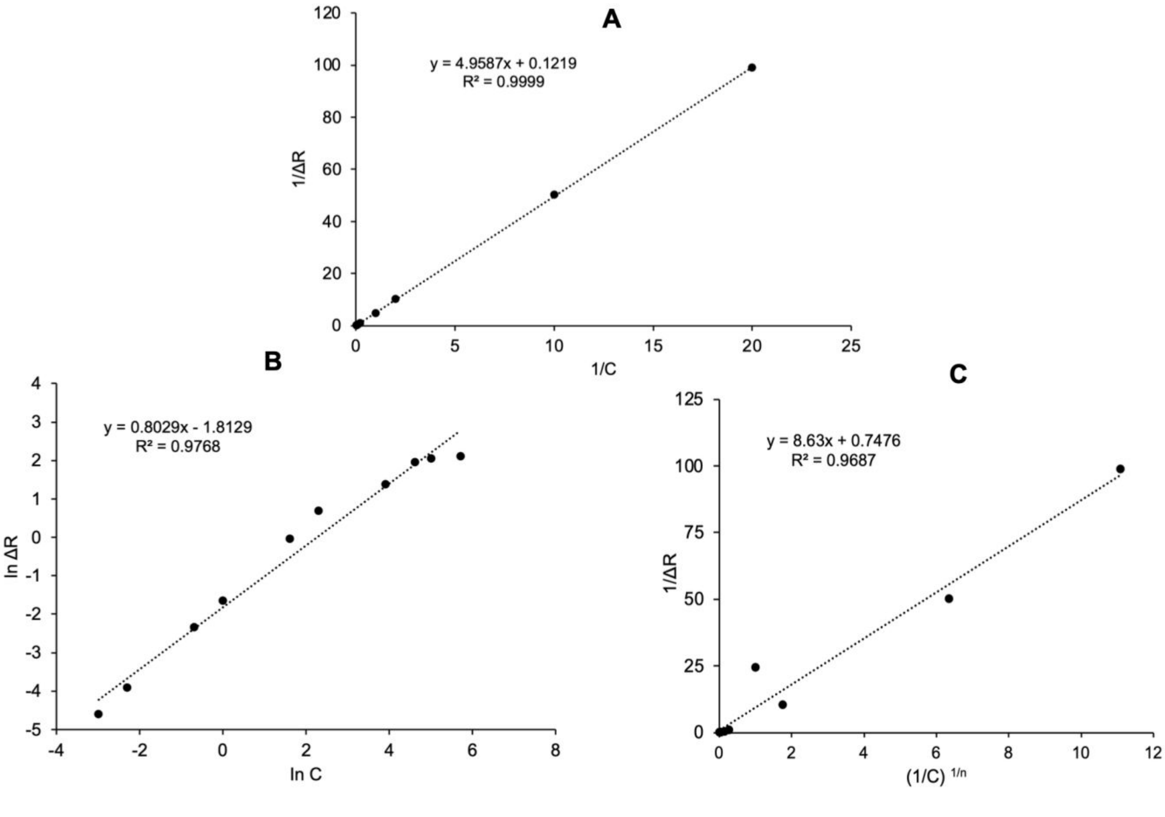

Figure S3. Kinetic Isotherm Models (Langmuir (A), Freundlich (B) and Langmuir-Freundlich (C).

TABLE S2. Isotherm Parameters of MIP SPR Sensor.

**Langmuir Isotherm**	**Freundlich Isotherm**	**Langmuir-Freundlich Isotherm**
$\Delta R_{max:}$ 8.203	ΔR_{max}: 6.128	ΔR_{max}: 1.337
K_A, $(ng/mL)^{-1}$: 0.024	1/n: 0.8029	1/n: 0.8029
K_D, ng/mL: 40.67	R^2: 0.9768	K_A, $(ng/mL)^{-1}$: 0.086
R^2: 0.999		K_D, ng/mL: 11.54
		R^2: 0.9687
